# The Hypodermic Treatment of Syphilis

**Published:** 1883-05

**Authors:** A. Lagorio

**Affiliations:** Chicago, Illinois


### ARTICLE II.

THE HYPODERMIC TREATMENT OF SYPHILIS. By A. LAGORIO, M.D., Chicago, Illinois. (Read before the Chicago Pathological Society.)

Syphilis is a virulent disease which never develops spontaneously, but is transmitted by a mediate or immediate contagion, by inoculation or by heredity. As all other virus, so the syphilitic cannot be known except by its effects, it not presenting any chemical nor special histological characters. Syphilis, once contracted, impresses on the organism a special and diathetic state, which is shown by various manifestations, which may be protracted to distant epochs from the moment of its infection. From the periphery of the body the disease proceeds toward the central parts; at first limited to the skin and mucous membranes; afterward invading the cellular tissues, the muscles, the bones and the viscera.

Although the venereal contact is the most frequent origin of syphilis, it is frequently acquired by many other means, as by kissing, by nursing, by smoking, etc., etc. It is only sufficient that the virulent products of a syphilitic individual come in contact with a sound individual who has the least solution of continuity of the skin or of the mucous membrane.

Guaiacum was once the great remedy for syphilis. Then the *cura famis*, or the *penitential Lent*, was established, consisting in the use of guaiacum and the privation of food, but the victims of this treatment soon made the method fall into discredit. Sudoriferous woods were then tried, as sarsaparilla, sassafras, etc., etc., and the preparations of gold and the mineral acids gained great favor. Berengarius da Carpi, having noticed a certain analogy between lepra and the cutaneous forms of syphilis, thought to cure them with mercury, and the results immensely surpassed his expectations. In lepra the remedy acted only as palliative, but it perfectly cured the syphilitic forms. He always obtained the most brilliant results; he became a great celebrity, and all ran to the Ligorian shore to regain the lost health. He became immensely rich, and to such an extent, that it was believed he had truly found the Philosopher's Stone, and the method so much sought by the alchemist, to transform any metal into gold. After this time every one applied himself seriously to study; experiments were made, one after the other, and the metal mercury passed into the scientific period. It seems that in the beginning of the seventeenth century, Paracelsus began to experiment on the action of mercury in syphilis, but the enormous doses which he used in a brief space of time, and his method of anointing the body in heated rooms, and the great number of mercurial poisonings, aroused against him the most learned physicians, who showed the bad effects of the method, and the remedy was abandoned. It was not until a century and a half later that the mercurial preparations began to be used with more care, in moderate doses, and with due intervals between the applications. Then it was known that salivation was not necessary for the efficacy of the cure, and that this depended upon the curative power of the remedy, and not from the secretion and excretion produced by the remedy. Along with this treatment, the use of wine, of meat, of free exercise in open air, was permitted to the patients, and many other things which before were thought to be injurious to the good end of the therapeutical treatment.

Later, the distinction between venereal and syphilitic affections, which before were treated promiscuously, served greatly to confirm the utility of mercury; but after so many experiments, after so many millions of patients who served as proof, the bad feeling that many have against this remedy has not yet ceased, and yet a remedy has not been discovered more capable of antagonizing the syphilitic contagion when once it has penetrated the system. Carbolic acid, salicylic acid, thymol, pilocarpine, iodoform, tayuya, and many other remedies, have been recommended in these later times, but the chief credit has always remained to mercury.

Mercury is used internally and externally; externally, in the form of baths, fumigations and frictions; internally, by inhalation, by the stomach, by the rectum, and lastly, by hypodermic injection.

When baths are used, the sublimate is generally preferred, at a dose of two to ten grams in a common bath. It is very useful, especially in infantile syphilis, and care must be taken to use it only when the skin does not present any ulceration, nor any solution of continuity capable of promoting the absorption. The patient remains in the bath from an hour to an hour and a half, and it is repeated every two or three days. In case of extensive serpiginous ulcers, or of softened and ulcerated gummata, disseminated in various regions, the dose of the sublimate in a bath must never be higher than two grams, as to act simply as a modifier of the eruptive form.

Fumigations, recognized since the sixteenth century by the Italian, Nicholas Massa, and now commended by Langston Parker, and by Paschkiss, of Vienna, consist in exposing the affected parts to the vapors of cinnabar, or to calomel, according to H. Lee. This method is especially adapted to those individuals possessed of a special constitution incapable of supporting mercury in any of its more active forms. Inunction is preferred in individuals unable to tolerate the remedy by the stomach, or by the hypodermic injection. But this plan of treatment is rarely regularly executed, because, as soon as the patient feels a little bettering of his sufferings, he will discontinue the tedious and uncleanly frictions, and stop the treatment.

The inhalations of sublimate do not act advantageously on general syphilis, but possess a curative value in some affections of the larynx and pharynx. The method most commonly used in the administration of the remedy by the stomach. Mercury is used by the stomach in those cases where, from the exceeding delicacy of the skin, the mercurial ointment causes inflammatory and vesicular eruptions of more or less extension, and where particular reasons prohibit the method of inunction. It is well known, and we must not forget, how mercury irritates and tires the stomach and enfeebles it; and in individuals badly organized, with morbid or hereditary dispositions, or affected with other diseases, the treatment often fails, and the syphilitic forms are reproduced, and become remittent and malignant. O'Connor proposed the introduction of mercury by the rectum in the form of suppositories. This method seemed once the surest; secret, easy, and of no annoyance to the patient. But practice has shown that only few patients could support it three or four weeks, and that almost all of them suffered with tenesmus, pains, and recto-intestinal catarrhs. Wallace made known the value of the potassic iodide. This can be given from the beginning to subdue the phenomena of syphilis, but it does not have any decisive action, except in the later forms.

Such a development as the hypodermic method has made in these few years in the treatment of syphilis, in defiance of the false and wrong interpretations of the various opinions, and of the disheartening reports from renowned authors, demonstrates that this method of administering medicines is a serious and a necessary practice of the clinical physician.

To Lewin belongs the merit of having introduced the hypodermic injections of mercury in the treatment of syphilis. He has largely used the method in the Charity Hospital of Berlin, and has obtained surprising results. From that time, this mode of treatment gradually generalized to such an extent, that to-day a physician of the large clinics of Europe is rarely found, who does not use this useful method to conquer and dispel the syphilitic manifestations, without the least injury to the stomach, in an energetic and eminently quick way.

In May, 1866, the *Lancet* contained a report of Dr. Berkely Hill, who had treated eleven individuals affected with constitutional syphilis by the hypodermic injections, made of a solution of corrosive sublimate. In four of the individuals, five centigr. of the salt were sufficient to determine hydrargirosis ; in other four seven centigr. were needed. The quantity used was one centigr. in each inoculation ; higher doses produced colics and diarrhœas ; moreover, the inoculated spot remained painful for some time. Only once he saw a pustule develop in the place of puncture. Scarenzio, professor of syphilis in the University of Pavia, was one of the first to make use of the hypodermic method after Lewin. He injected calomel at a dose of twenty cent., and once of thirty cent., with a gram of dist. water and one-half gram of mucilaginous liquid, and reported to have obtained rapid cures without salivation. Only in few cases he saw the development of a small abscess, which rapidly healed after incision. C. Manassei, professor of cutaneous diseases and of syphilis in the clinic of H. Gallicanus, Hospital of Rome, repeated the experiments of Scarenzio, but, knowing the inconveniences of the abscess which generally followed the inoculation of calomel, to prevent it, and to make more applicable the hypodermic method in private practice, thought to use small and often repeated doses of corrosive sublimate; so doing, he had it immediately absorbed, and avoided the cauterizing action of the same. But, fearing that the corrosive sublimate introduced under the skin would produce gangrene, he experimented at first in the rabbit, and having obtained good results without any inconveniences in the skin, he used it to a large extent in the treatment of many syphilitics, whose clinical histories yet remain in the archives of St. Spiritus Hospital. The daily dose was one centigram in diluted solution, divided in two injections in different localities.

Dr. E. Rasori, assistant to the clinic in the Hospital of Venereal Diseases, of Rome, reports * to have treated, up to December, 1879, forty-six patients affected with syphilis with the hypodermic injections. He accurately followed the course of the disease, experimented on the action of the remedy, and constantly kept them under observation up to 1881, so as to be able to notice any return of the disease, if happening. Of these, he reports 38 completely cured, and eight he denotes as ameliorated, for they were lost sight of, although at the time of dismissal from his care he considered them as cured. Of these, 29 were men, and 17 women. The number of injections used in each patient were 25 to 30. Only in three cases he resorted to calomel injections; the balance were treated with the sublimate.

Martineau has reported over 170 cases treated with the injections. He prefers the peptonate of mercury, and believes that his method of treatment acts more promptly and energetically upon syphilitic manifestations than any other.

Grefberg has also reported a large number of patients treated with the injections. He used the bi-cyanuret of mercury with little morphine. His results have been good.

Professor Semmola, of Naples, has also reported quite a number of syphilitics cured with the subcutaneous injections. He injects one or two mill. at a time, and repeats the operations two or three times a day, without any local accidents. In 25 to 30 days he has cured the most confirmed cases of syphilis. He especially reported two cases, in whom he made 25 injections of ½ centigr. a day of the sublimate, with a complete cure. To avoid any local irritation, he adds to the sublimate a chloride and albumen. His formula is: Corrosive sublimate, 25 centigr.; sol. ammoniac, 3 grams; water, 30 grams, and to this he adds the white of an egg, which produces a clear liquid, somewhat dense, of which he generally takes two drops, which correspond to two milligr. of sublimate, and injects with a gram of distilled water. Prof. D'Amicis

* *Giornale Intern. delle Scienze Mediche.*

has also used to a large extent the corr. sublim. in diluted solutions, in hospitals and private practice, without the precaution of the chloride of ammonium and the albumen, as Prof. Semmola, and the injections were very well tolerated. Wrongly some have denied the prompt absorption of the mercury, inasmuch as Scarenzio and others, by introducing a catheter in Steno's duct, found in the saliva traces of calomel 24 hours after the injection of 20 cent. of calomel. The hypodermic method is sufficiently energetic. When a prompt improvement is needed for fear of grave morbid sequences, for instance, in case of iritis, with danger to the bulb and the visual faculty; in symptoms of cerebral compression, or in women in an advanced stage of pregnancy, with a possibility of an abortion or of near death of the fœtus; and in cases of desperate and malignant syphilis, it is of the utmost value to use corrosive subl. hypodermically; or, better, calomel with care to promptly open any abscess, if happening in the spot of inoculation.

I have seen in Rasori's clinic, with only one injection of 25 cent. of calomel suspended in 1½ grams of glycerine, a speedy bettering of an iritis within 24 hours, in a woman of feeble constitution, affected with tertiary syphilis; and also in two males who presented secondary manifestations of the disease. In the latter, one injection was sufficient, while in the former, two were needed. In a few days the photophobia, the pain and the redness rapidly diminished, and the pupil slowly regained its mobility. At the same time atropine was used locally, and the abscess, when opened, promptly healed, without any consequences.

A young woman twenty-six years old acquired a syphilitic infection. She applied to me in the month of April, 1881; she was then suffering with the secondary manifestation of the disease, and a marked bilateral iritis. The pain and the photophobia were intense; the pupil fixed, irregular, and with almost impossibility to fix upon any object. I injected in the left arm 20 centigr. of calomel in a gram of glycerine. Next day, the left eye showed a slight bettering, but the right one almost none. I then again made another injection, in the right arm. In 24 hours the bettering was marked in both eyes; a little more in the left. She stood on that day a prolonged examination. No more injections of calomel were made, but atropine was applied, and the bandage.

In about 20 days, the patient could use perfectly both eyes. The small abscesses produced by the injections were opened, and promptly healed. I finished the treatment of the syphilis with the sublimate injections. After 23 injections, every symptom disappeared, and after eight more, I pronounced the patient cured; in all 31 injections. Never saw the patient again until three months ago, when I learned she enjoyed good health, and thought to be in the second month of pregnancy.

The energy of the hypodermic treatment is also proven by the brevity of the time in which the symptoms disappear. In the greater number of cases which Rasori reports, after 20 injections, made alternately, the secondary symptoms of the diseases disappeared; in the worse cases, 30 to 40 injections were used, but rarely this occurred. He says that more than once has it occurred to him to see in a few days, as of wonder, the disappearance of symptoms which seemed permanent; such bettering rarely occurred in the ordinary method of treatment. Generally, the injections ought to be continued until, by palpation, we cannot any longer feel the indurated cervical glands. Only then we can feel sure of the complete cure. The determination of the complete cure of syphilis is one of the most difficult clinical tasks; many years of constant observation are necessary, not to the patient alone, but the observations must be carried to his offspring. Many authors assert that relapses are less frequent when the hypodermic method is used. During this treatment I have never seen a patient presenting any disturbance of the digestive or circulatory apparatus, nor in the function of the senses. In rare cases, traces of albumen may be found in the urine, which soon disappear, and never reach a notable amount. A progressive bettering and a better aspect of the patient is noted, and we feel sure that under the action of the treatment, the disease is sensibly ameliorated. The action of the remedy is very often shown in the salivary glands, although in many cases remaining unobserved; the patient feels only a slight metallic taste, of which he most frequently becomes aware when he is questioned about it; therefore, instead of being a true sensation, it may be only a mere fancy. Sometimes a little redness appears along the border of the gums around the teeth, especially if these are decayed. But if the mouth and the fauces are regularly kept clean and in good condition, we can almost remain assured that the worst forms of syphilis can be cured without the least salivation.

The solution most commonly used by Rasori is: ℞. Corrosive sublimate, 1 gram; distilled water, 100 grams; chloride of sodium, 1.50 grams. This, filtered and bottled, will keep for some time. The addition of the chloride of sodium is found to be of great value, inasmuch as with it the amount of coagulation and infiltration in the point of injection is less, and the absorption is considerably increased; this is explained by the more easy and rapid solubility of the albumen precipitated by the sublimate in the connective tissue. It is a rule never to use more than one gram at each injection. The calomel is used suspended in glycerine, at a dose of 30 centigr. in two grams of liquid. If this dose is used, an abscess will follow, while by injecting 5 centigr., no notable reaction occurs in the spot of puncture. This mixture must be prepared always before using. Sigmund affirms to have obtained more favorable curative results with small doses of calomel (5 to 10 cent.) than with higher ones. According to his assertions, in many cases, not only he failed to see the abscess, but also the pain.

The cutaneous injection, considered as an operation, is most simple, and does not present any important singularity. Glass syringes, mounted in hardened rubber, are to be preferred to metal ones. The operation is conducted in the same manner as is every day done with any hypodermic syringe.

Ordinarily, the injection produces no pain in the act of operation; in some cases, the part becomes somewhat painful, but the pain easily ceases on the application of wet compresses. The addition of morphine to the liquid is not necessary. When the skin is delicate and sensitive, there remains a small circumscribed indolent induration, the shape of a node, which lasts some little time, around the puncture, and if another injection is attempted in the same place, the liquid will not penetrate, but regurgitate. From this, the rule not to make the punctures too near to each other.

The gluteal and dorsal region is generally preferred for the thickness of the skin, and for the little pain produced by the movements of the body. In worse cases, where it is necessary to act rapidly, and calomel is used, the injections are better to be made in the arm, at the insertion of the deltoid and biceps, where any abscess which may be forming can be easily emptied and treated. The formation of an abscess is the greatest charge against the hypodermic method, but in the greatest number of cases, it is mainly due to the rough manner of the operator, and to the too little attention paid by him. One thing which is seldom recommended sufficiently, is to keep the syringe clean; it must be washed and diligently dried. It is unnecessary to say that it must be used only for syphilitic patients.

The syphilitic manifestations are easily disturbed, made worse, and rapidly changed by external influences that act unfavorably on them; therefore, hygiene is of utmost importance in the treatment of syphilitics. The patient must seek uniformity in the mode of living. Moving, exercising and attending to daily duties are not to be avoided, unless done in excess. In stormy weather, in-doors is necessary, and also a uniform temperature of the room, renovating frequently the air, without any exposure to any current, to avoid taking cold. The patient will keep to his room and bed in case of a general eruption of symptoms, or during an energetic treatment. The obstinacy of the disease frequently makes a patient despondent, downhearted, and lose confidence in the treatment; here the authority of the physician will do great good.

The diet should consist of such food as is easily digested, nourishing, and not stimulating. Meat, milk, eggs, light vegetables and starchy food is to be preferred daily; while aromatic and compounded drinks, vegetables, fats and alcoholics are to be avoided. Smoking is also to be avoided, especially if the oral mucous membrane is diseased. The irritation of the smoke interferes with the healing of the local lesions of the mouth long after the disappearance of the symptoms, just in the same manner that rough work will produce identical results in the psoriasis of the hands, and scratching in the acneiform eruptions of the face.

Tonics and ferruginous preparations are of great benefit in the feeble, lymphatic, in the marasmus of syphilis, and in tardy manifestations of the disease. Marine air, and living in thermal and mountainous regions, are of benefit.

In the gummous tertiary forms, the decoction of Zittman has lately been highly praised, and was largely used by Hebra. Besides such substances as alum, sugar, cinnabar, senna, etc., its action is mainly due to sarsaparilla and calomel. The treatment and the hygienic rules are to be followed for some time after all symptoms have disappeared, to avoid any returns.

I have had occasion to treat some syphilitics with the methods that I have endeavored to explain to you in this paper. I would relate their histories, methods of treatment adopted, and results, but I fear I have already delayed myself too long. My expectations with the hypodermic injections of mercury in the treatment of syphilis have been successfully fulfilled, with great satisfaction to myself and happiness to my patients.

From all that I have so far said in a brief and imperfect manner, I wish not to deduce that the injections constitute the only sure method to conquer all the stages and morbid phenomena of syphilis, nor that they are to be preferred to any other therapeutical method. My intent is only to make known their utility, and the convenience that they afford in public institutions; the little time lost by the patient and the physician; the brief inconveniences that they cause; the little or no danger of stomatitis and of mercurial salivation; the little disturbance to the stomach; the secrecy with which a treatment can be continued; the satisfaction to be able to determine the exact dose of mercury introduced in the organism, and the certainty of the exact continuance of the curative method, which in other ways depends upon the caprices, the business, or the ideas of the patient; and moreover, it is regulated by the constant watch of the physician.

In the greater number of cases, an energetic treatment is effected with the injections, which, without any notable disturbances and bother, bring to a rapid and durable cure, rarely followed by any returns; a result that we can hardly hope to obtain by adopting other methods.

